# The Effect of Plasma Treated Water Unit Processes on the Food Quality Characteristics of Fresh-Cut Endive

**DOI:** 10.3389/fnut.2020.627483

**Published:** 2021-01-27

**Authors:** Uta Schnabel, Oliver Handorf, Hauke Winter, Thomas Weihe, Christoph Weit, Jan Schäfer, Jörg Stachowiak, Daniela Boehm, Harald Below, Paula Bourke, Jörg Ehlbeck

**Affiliations:** ^1^Leibniz Institute for Plasma Science and Technology, Greifswald, Germany; ^2^School of Food Science and Environmental Health, Technological University Dublin, Dublin, Ireland; ^3^Institute for Hygiene and Environmental Medicine, University Medicine Greifswald, Greifswald, Germany; ^4^School of Biosystems and Food Engineering, University College Dublin, Dublin, Ireland; ^5^School of Biological Science, Institute for Global Food Security, Queens University Belfast, Belfast, Northern Ireland

**Keywords:** atmospheric pressure plasma, food quality, leafy greens, microwave-driven discharge, non-thermal processing, ready-to-eat produce

## Abstract

This study evaluated the impact of a defined plasma treated water (PTW) when applied to various stages within fresh-cut endive processing. The quality characteristic responses were investigated to establish the impact of the PTW unit processes and where PTW may be optimally applied in a model process line to retain or improve produce quality. Different stages of application of PTW within the washing process were investigated and compared to tap water and chlorine dioxide. Fresh-cut endive (*Cichorium endivia* L.) samples were analyzed for retention of food quality characteristics. Measurements included color, texture, and nitrate quantification. Effects on tissue surface and cell organelles were observed through scanning electron and atomic force microscopy. Overall, the endive quality characteristics were retained by incorporating PTW in the washing process. Furthermore, promising results for color and texture characteristics were observed, which were supported by the microscopic assays of the vegetal tissue. While ion chromatography detected high concentrations of nitrite and nitrate in PTW, these did not affect the nitrate concentration of the lettuce tissue post-processing and were below the concentrations within EU regulations. These results provide a pathway to scale up the industrial application of PTW to improve and retain quality characteristic retention of fresh leafy products, whilst also harnessing the plasma functionalized water as a process intervention for reducing microbial load at multiple points, whether on the food surface, within the process water or on food-processing surfaces.

## Introduction

As with many other vegetables, fresh-cut lettuce (e.g., endive) is a minimally processed produce that is harvested, cut, washed, centrifuged, and packaged (1, 2). These activities may be associated with mechanical damage to plant tissue, which causes biochemical and physiological reactions such as enzymatic browning, increased respiration, sensory, and structural decay (3). These changes may lead to significant losses in quality and thus reduce the shelf life and marketability of the produce (4, 5). Washing the fresh-cut lettuce is used to remove field heat, dirt, microorganisms, possible pesticide residues, and cell exudates, which could otherwise lead to a loss of quality (6, 7). Washing is therefore particularly important for the microbial safety and storage quality of fresh cut lettuce.

However, the process water can also be a source of microorganisms and lead to cross contamination. Therefore, where the use is legally permitted, water additives, mostly chemical, are used to reduce the microbial load in the washing water. Chlorine-based compounds are the most common and widely used disinfectant (8, 9). However, the use of chemicals is not permitted in the production of organic food and in conventional food processing the use of chemical disinfectants is not without concern, as they can lead to the formation of potentially harmful haloform by-products namely chloramines and trihalomethanes (10). Other wash water additives or treatments already in industrial use include chemical sanitizers like ozone, hydrogen peroxide, electrolyzed water, and peracetic acid. Physical treatments such as high hydrostatic pressure, pulsed electric field, oscillating magnetic field, ultra violet (UV)- or gamma irradiation and high-power ultrasound are also possible (4, 11–15). Some innovative process water additives under research are Quillaja saponaria extract (QSE) and Nα-lauroyl-L-arginine ethyl ester (LAE) (16–18).

The development of sustainable disinfection methods is important and challenging, but product quality compatibility, cost, environmental impact, and regulatory must also be met (19). An innovative strategy under research to reduce the bacterial load of process water and subsequently, to keep the food quality and shelf-life of fresh-cut lettuce at high levels is the use of plasma treated water (PTW) as an antimicrobial process stage.

The application of non-thermal plasma (NTP) generated at atmospheric pressure is a promising physical approach (20, 21). Plasmas are ionized gases containing neutral- and free charged particles such as ions and electrons (22, 23). Novel intervention technologies for fresh foods demand minimal processing at low or mild heat temperatures to maintain fresh characteristics, as well as compatibility with high throughput continuous processing, which can be achieved through non-thermal plasma at atmospheric pressure in gas or functionalized liquid mode of delivery (22). PTW can be used as the transport medium of reactive species and antimicrobial components for food, water, and surface sanitation (24). PTW is comparable to ozonized or chlorinated water with regard to mode of application and antimicrobial effects. The chemical composition of PTW concerning the acidic pH, and the reactive oxygen and nitrogen species (RONS) was previously characterized (25). Both, low pH and RONS are known to support and to cause the antimicrobial mechanisms of action, therefore the chemical composition of PTW should be known if PTW is investigated as a process wash water. This study investigates the unexplored aspects of how the PTW effects the food quality of fresh-cut endive and determines where PTW may be optimally applied within a fresh produce washing process for produce quality retention.

## Materials and Methods

### Generation of PTW and Its Chemical Characterization

The PTW generation was previously described in (25). In brief, plasma processed air (PPA) was used to treat distilled water. This leads to the formation of PTW. The used plasma source was a two-stage microwave-driven device (2.45 GHz) based on a single-stage plasma torch (26, 27) and operated at atmospheric pressure. The used technical parameters for the presented experiments to generate PPA and subsequently PTW were 1.3 kW (power) and 12 slm (volume flowrate) for the first stage. For the second stage, 3.0 kW and 60 slm were applied. The chemical composition of PPA and PTW was previously reported (25, 28). Briefly, using emission spectroscopy (ES) analysis, the spectral lines of nitrogen monoxide radical (·NO), nitronium cation (${\text{NO}}_{2}^{+}$), and hydroxide anion (OH^−^) were dominant in the detected spectrum (28). In the FTIR analysis, the main components of PPA were nitrogen monoxide radicals (·NO), nitrogen dioxide radicals (·NO_2_) and water (H_2_O), oxygen (O_2_) and nitrogen (N_2_). ·NO was determined with a concentration of 2,900 ppm (7.79 × 10^22^ m^−3^), ·NO_2_ with 76 ppm (2.04 × 10^21^ m^−3^) and H_2_O with 9,200 ppm (2.47 × 10^23^ m^−3^) (25). The chronoamperometry identified a H_2_O_2_ concentration of 5.61 mg L^−1^ (29.39 mM) in the PTW (25). Finally, the ion chromatography (IC) measurements identified high values for ${\text{NO}}_{2}^{-}$ and ${\text{NO}}_{3}^{-}$, 687 mg L^−1^ ${\text{NO}}_{2}^{-}$, and 1,227 mg L^−1^ ${\text{NO}}_{3}^{-}$, respectively (25).

### Investigated Specimen—Fresh-Cut Lettuce With Native Load

The specimen (endive, *Cichorium endivia* L.) was bought at a local organic market in Greifswald, Germany. The lettuce was grown on different fields (loamy sand and sandy loam) in the state of Mecklenburg-Western Pomerania, Germany. The harvest months were October to December 2019. Subsequently, the whole endive heads were stored in the dark for a maximum of 24 h at 7.4 ± 0.1°C before use. The relative humidity inside the fridge was 78% to 99% with a dew point of 3.9 td to 7.3°C td. Produce samples were prepared in accordance with previous studies aligned to industry practice and are briefly presented here (25). For food color and texture analyses, the lettuce was cut before washing. In the case of the microscopy analysis, the lettuce was washed first and subsequently cut. Before experimental use, the outermost leaves were removed, but the stalk was retained. Both, the softer leaf parts and the harder stalk parts of the lettuce were mixed to provide representative. Prepared experimental samples were stored in closed homogenizer bags (polyethylene; VWR International GmbH, Darmstadt, Germany) in air and removed for analyses ay days 1 and 7.

### Processing of Fresh-Cut Endive

**Figure 1:**
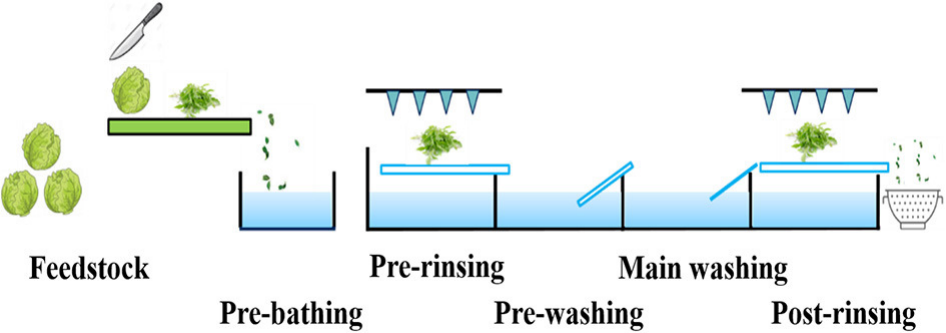
An example process line, the illustrated washing process of a tap water reference (process variant Ia): five washing sections—pre-bathing, pre-rinsing, pre-washing, main washing, and post-rinsing.

The processing of the fresh-cut endive was performed on a washing line mimicking a common industrial production process. The washing line ultimately consisted of up to five main sections—pre-bathing, pre-rinsing, pre-washing, main washing, and post-rinsing. The investigated process variants are given in Table 1 and as an example, the reference process using tap water is illustrated in Figure 1. After the last washing step, the samples were placed over a sieve for draining, but were not spun.

**Table 1:** Investigated process variants included the application of PTW at various process stages and unwashed lettuce, tap water and chlorine dioxide (ClO_2_, 15 ppm) were the reference treatments.

**Process variant**	**Unwashed** **(0)**	**Pre-bathing** **(1)**	**Pre-rinsing** **(2)**	**Pre-washing** **(3)**	**Main washing** **(4)**	**Post-rinsing** **(5)**
Time of washing in [s]	0	180	30	180	180	30
Ia—tap water	NA	**Tap water**	**Tap water**	**Tap water**	**Tap water**	**Tap water**
Ib—tap water	NA	NA	**Tap water**	**Tap water**	**Tap water**	**Tap water**
II—PTW	NA	**PTW**	**Tap water**	**Tap water**	**Tap water**	**Tap water**
III—PTW	NA	NA	**PTW**	**Tap water**	**Tap water**	**Tap water**
IV—PTW	NA	NA	**Tap water**	**Tap water**	**PTW**	**Tap water**
V—PTW	NA	**PTW**	**PTW**	**Tap water**	**PTW**	**Tap water**
VI—ClO_2_	NA	NA	**Tap water**	**Tap water**	**ClO**_**2**_	**Tap water**

*The washing time for each of pre-bathing, pre-washing and main washing was 180 s and the washing times for pre- and post-rinsing were 30 s. NA, not analyzed. For better visualization, tap water treatment is highlighted in blue, PTW treatment in pink, and ClO_2_ treatment in red*.

### Characterizing the Impact of Process Variants on Food Quality Parameters

#### Color Analyses

Color analyses on lettuce leaves were performed using a portable colorimeter NH310 of 3nh (PCE Deutschland GmbH, Meschede, Germany) with the CIELab system. Five points of measurement were used for each lettuce leaf, and chroma was expressed as *C*-value. For the process variants listed in Table 1, the Chroma was examined after each washing section. These investigations were carried out immediately on the day of treatment (day 0), 24 h (day 1), and 168 h (day 7) later.

#### Texture Analyses

The texture of the fresh-cut endive samples was examined with the Texture Analyser TAXT+ (WINOPAL Forschungsbedarf GmbH, Elze, Germany) before and after treatment with tap water, PTW, or ClO_2_. Samples had a mass of 10 ± 2 g. Five samples were examined for each process variant, washing section, and storage time after treatment (0, 1, and 7 days). The sample to be measured was transferred into a 600 mL beaker and positioned under the probe head. A probe head with three concentric rings (WINOPAL Forschungsbedarf GmbH, Elze, Germany) was used, as the large cross section is appropriate to measure the impact of the force over the whole leaf samples structure. The used protocol was previously described in Schnabel et al. (25).

#### Determination of Nitrate (${\text{NO}}_{3}^{-}$) Content

Before and after washing the fresh-cut lettuce with tap water, PTW and ClO_2_, the plant tissue was homogenized with a common hand blender. After blending, 5 g of the homogenized plant tissue was mixed with 50 mL sterile tap water (70°C), briefly shaken and incubated for 15 min. Two filtration steps were completed after incubation. For the first step, the whole tissue sample was rinsed over a paper filter (VWR, Darmstadt, Germany; particle retention of 2–3 μm). In the second step, the filtrated solution was filtrated again by a tip filter [Sarstedt, Nümbrecht, Germany; particle retention 0.2 μm, PES (polyester) membrane]. The collected double-filtrated solution was analyzed by IC for ${\text{NO}}_{3}^{-}$ concentrations according to the following procedure. Immediately after sample preparation, nitrate was determined by ion chromatography (IC). For this purpose, the IC was performed with the 850 Professional IC (Deutsche METROHM GmbH & Co. KG, Filderstadt, Germany) as previously described in detail in our publication Schnabel et al. (25).

#### SEM

Fresh samples of fresh-cut endive with an area of 25 mm^2^ were retrieved before and after treatment with tap water, PTW and ClO_2_. They were then prepared on brass holders with an electrically conductive glue containing silver particles (Ferro GmbH, Germany). The samples were dried under vacuum (1.0 mPa, 24 h) and subsequently coated with thin gold film by the sputter coater SCD 050 (Bal-Tec, Switzerland) in order to adjust the optimal material conditions for observation by means of an electron microscope. Overview and high-resolution images of the samples were taken with the electron microscope JSM 7500F (Jeol, Germany) at magnifications 100; 1,000; and 4,000. A secondary electron detector with a resolution of 1.0 nm was used for this morphological analysis. The microscope setting in this study was applied as follows: accelerating voltage 1 kV, working distance 8 mm, samples surface in the perpendicular position to the beam.

#### AFM

A fresh-cut lettuce tissue sample was fixed onto a PE-holder (32 × 8 × 2 mm^3^), and placed in a 60 mm diameter petri dish. Following a standard procedure (25), the sample have been submerged in 5 ml of filtered tap water (tip filter, which excludes all particles >2 μm) and fixed beneath the measuring head of the AFM. We used a NanoWizard III (JPK BioAFM, Bruker, Berlin, Germany) with a linearized piezo scanner for the scanning-probe topographies. Therefore, the scanning covered a travel path length of the piezos of 100 μm in every direction (*xyz*). Beam-shaped silicon probes without any top coating, a nominal spring constant of 0.29 N m^−1^, and a pyramidal-shaped tip (nominal aspect ratio: 1.5–3.0) were used. All samples were measured in contact mode with a set point of 15 nN and a line rate of 0.08 Hz by a 90 × 90 μm^2^ scan width. Two recording modes were used; the height micrograph reflects the vertical (*z*-information) extension of the piezo and the horizontal movement of the lateral scanner whilst it scans over the surface. It reflects the exact topographic height differences on the entire scan area. The deflection image, which is also referred as the error signal, reflects the deflection, i.e., the bending, of the probe that has been plotted against its *xyz*-position.

#### TEM

Transmission-electron microscopic (TEM) micrographs were taken before and after each washing step with tap water, PTW, or ClO_2_. Using a sterile razor blade, samples of a 5 × 20 mm^2^-area (5 mm length and a width of maximum 1 mm) were cut from green and healthy lettuce leaves. These pieces were fixed at room temperature for 2 h with a fixative containing 3% glutaraldehyde in a 50 mM cocadylate buffer at pH 7, and stored at 4°C until further processing. Before placing the samples in the high vacuum of the TEM, they were washed for 5, 10, 15, 20, and 30 min, and then embedded in low gelling agarose, further washed with cacodylate buffer for 10 min, prior to fixing in 2% osmium tetroxide (which was dissolved in cocadylate buffer) for 2 h at room temperature. Final samples were washed repeatedly as described above. For dehydration, the specimens were submerged for 15 min in distinct ethanolic solutions with increasing concentrations of 30, 50, 70, and 90%. Finally, we placed the samples in 96% ethanol for duplicate 10 min periods, and then in 100% ethanol thrice. In applying the flat-embedding technique, we transferred the samples stepwise into propylene oxide before they have been ingrained in low-viscous agar-resin (AGAR-LV, plano, Wetzlar, Germany). Subsequently, sections were cut on an ultramicrotome (Reichert Ultracut, Leica UK Ltd., Milton Keynes, UK), transferred onto pioloform-coated slot grids (2 × 1 mm, plano, Wetzlar, Germany), and stained with 4% aqueous uranyl acetate for 5 min. This was followed by lead citrate incubation for 1 min. The samples were analyzed with a TEM LEO 906 (Zeiss, Oberkochen, Germany) at an acceleration voltage of 80 kV, and micrographs were edited with Adobe Photoshop CS6 (Adobe Systems Inc., San Jose, Cal., US).

### Statistics

Our experiments aim to mimic an industrial process as close as it is possible and therefore the leaves are not treated the same way at every extraction point (EP) along the washing process (process variant I–VI). Some process variants (Ib, III, IV, and VI) miss extraction point 1 (pre-bathing). Consequently, no data were obtained for these aspects at EP1, which make a more direct testing based on a distinct hypothesis complicated. Additionally, tests need to be designed to test the data against two references. The colorfulness (C) of the fresh leaves and their texture (T) are basic values, which are used in our hypotheses. We assumed a gaussian distribution among the values of C and T. Consequently, the arithmetic means and a standard deviation were computed and used in a statistical test series. We tested against a statistical significance of α = 0.05.

Certainly, it is straightforward to assume that possible changes in C and T appear when the leaves are treated with different sanitizers, which is best observed at EP5. Therefore, we formulate the basic α-hypothesis:

$H_{0}^{\alpha }=$ The C or the T of the leaves did not change at EP5,

and the alternative:

$H_{1}^{\alpha }=$ The C or the T of the leaves changed at EP5.

For that reason, we tested C and T at EP5 with an ANOVA against the outcome of process variant Ia/b (tap water). By this way, a statistical evaluation of possible differences in C and T of treated leaves based on a *p*-value that mirrors the probability of error for an incorrect rejection of the null hypothesis at that point was possible. A second test series evaluated every single step of each process variant against the input-values (EP0) of C and T based on ANOVA. By this way, a statistical evaluation of possible C and T changes along every process variant was possible. Therefore, the β-hypothesis is formulated:

$H_{0}^{\beta }=$ The C or the T of the leaves do not change due to the treatment and the washing stage along a given process variant,

and the alternative:

$H_{1}^{\beta }=$ The C or the T of the leaves changed due to the treatment and washing stage along a given process variant.

In summary, we have a set of horizontal ANOVA-tests for every process variant (I–VI, β-test) and a vertical ANOVA-test at EP5 (α-test). The β-test evaluated whether any C or T changes occurred along a process variant. The α-test evaluated possible C and T changes at EP5. Combined, α and β revealed information about the experiment of a completely examined day (0, 1, and 7). Set the case, the input of leaves was, regarding their C and T, homogenous and the outcome at EP5 differs, changes were a consequence to a sanitizer treatment along a process variant. A behavior, which could be observed when the α-H_1_-hypothesis went through and if at least one β-H_0_-hypothesis was ineligible. Generally, we interpreted the *p*-values of the α- and β-tests for every examination day as strong indicators for the behavior of the daily experimental set up. Nevertheless, they alone give not a basis to test a hypothesis addressing the overall interpretation of a completely examined day.

The hypothesis addressing the changes in C and T over a time of 0, 1, and 7 days compared the mean values of the outcomes at EP5 against each other based on an ANOVA evaluation and the hypothesis:

*H*_0_= The C or the T of the leaves did not change over the time of 0, 1, or 7 days,

and the alternative:

*H*_1_= The C and the T of the leaves changed over the time of 0, 1, or 7 days.

That hypothesis allowed statistical statements about changes in C and T when the samples were stored up to 7 days.

## Results

### Color

**Figure 2:**
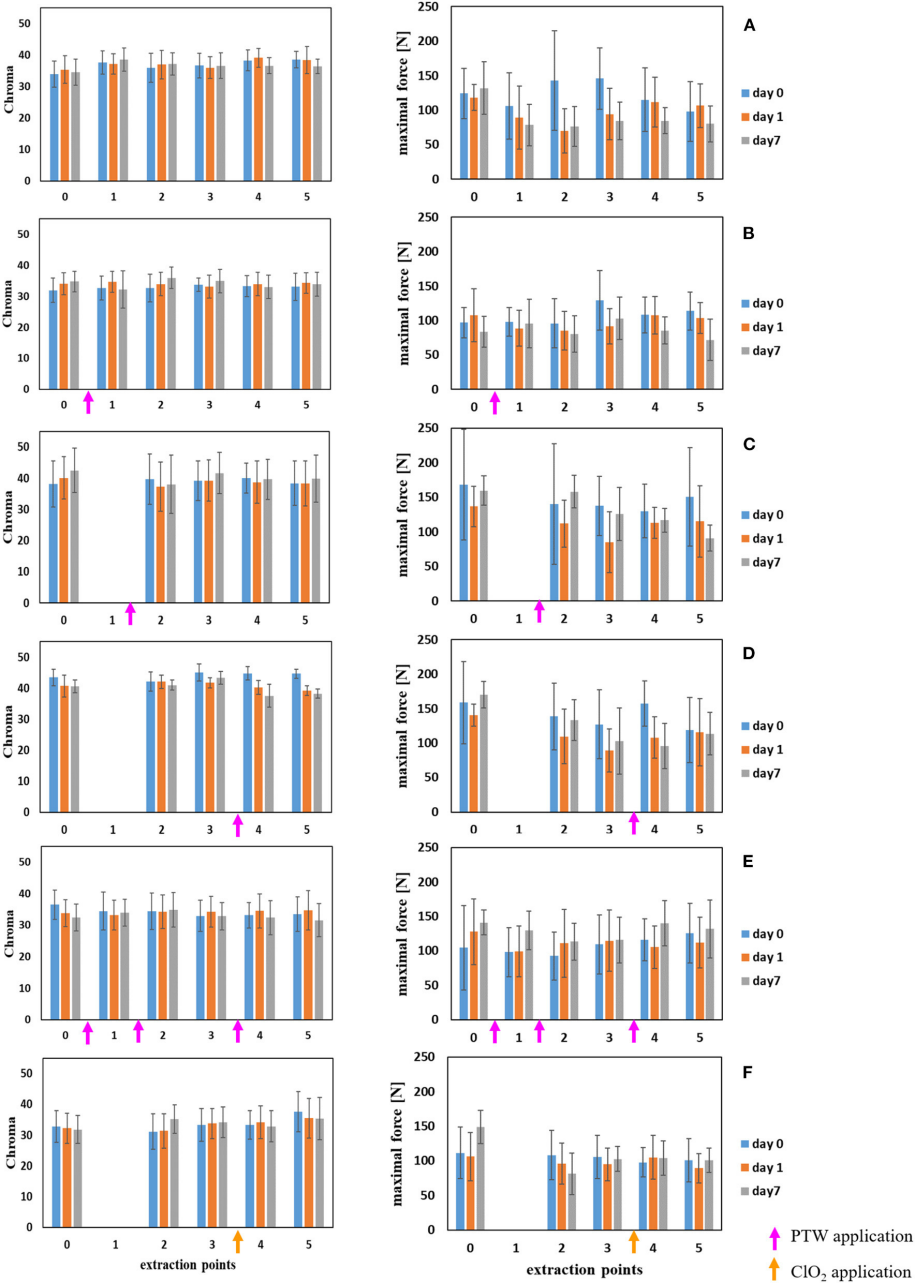
The effect of unit process treatments on color and texture stability of fresh cut endive. Color (left column) and texture measurements (right column) of unwashed (extraction point 0) and tap water, PTW or ClO_2_ washed fresh-cut endive (extraction points 1–5). **(A)** Variant I: tap water at all extraction points for storage days 0, 1 and 7. **(B)** Variant II: PTW at extraction point 1 (180 s pre-bathing), extraction points 2–5 with tap water for storage days 0, 1, and 7. **(C)** Variant III: PTW at extraction point 2 (30 s pre-rinsing), extraction points 3–5 with tap water for storage days 0, 1, and 7. **(D)** Variant IV: PTW at extraction point 4 (180 s main washing), extraction points 2, 3, and 5 with tap water for storage days 0, 1, and 7. **(E)** Variant V: PTW at extraction point 1, 2, and 4 (180 s pre-bathing, 30 s pre-rinsing, and 180 s main washing), extraction points 3 and 5 with tap water for storage days 0, 1, and 7. **(F)** Variant VI: ClO_2_ at concentration of 15 ppm at extraction point 4 (180 s main washing), extraction points 2, 3, and 5 with tap water for storage days 0, 1, and 7. All experiments were repeated threefold with *n* = 5 resulting in *n* = 15.

The results for the color analyses are shown in Figure 2 (left column). The change of green lettuce color after washing with tap water, PTW and ClO_2_ was determined directly (day 0), after 1 day and 7 days of storage at 7°C in a closed plastic bag. The Chroma values of the color measurements were not affected by any of the treatments studied within the 7-day storage trial. Statistical analysis revealed no significant color changes for every process variant and analysis day. The results of the statistical evaluation are given in Tables 2–5.

**Table 2:** Statistical evaluation of storage day 0.

**Process variant**	***p*-value**	***H*_**0**_ hypothesis**
Horizontal β evaluation		
I—tap water	0.59	Not rejected
II—PTW	0.12	Not rejected
III—PTW	0.75	Not rejected
IV—PTW	0.95	Not rejected
V—PTW	0.84	Not rejected
VI—ClO_2_	0.32	Not rejected
Vertical α evaluation		
Extraction point 5 (EP5)	0.09	Not rejected

**Table 3:** Statistical evaluation of storage day 1.

**Process variant**	***p*-value**	***H*_**0**_ hypothesis**
Horizontal β evaluation		
I—tap water	0.22	Not rejected
II—PTW	0.22	Not rejected
III—PTW	0.85	Not rejected
IV—PTW	0.90	Not rejected
V—PTW	0.90	Not rejected
VI—ClO_2_	0.39	Not rejected
Vertical α evaluation		
Extraction point 5 (EP5)	0.32	Not rejected

**Table 4:** Statistical evaluation of storage day 7.

**Process variant**	***p*-value**	***H*_**0**_ hypothesis**
Horizontal β evaluation		
I—tap water	0.17	Not rejected
II—PTW	0.44	Not rejected
III—PTW	0.86	Not rejected
IV—PTW	0.96	Not rejected
V—PTW	0.42	Not rejected
VI—ClO_2_	0.69	Not rejected
Vertical α evaluation		
Extraction point 5 (EP5)	0.12	Not rejected

**Table 5:** Statistical evaluation over all storage days (day 0, 1, and 7) at EP5.

**Process variant**	***p*-value**	***H*_**0**_ hypothesis**
ANOVA evaluation over day 0, 1, and 7 at EP5		
I—tap water	0.53	Not rejected
II—PTW	0.94	Not rejected
III—PTW	0.88	Not rejected
IV—PTW	0.97	Not rejected
V—PTW	0.94	Not rejected
VI—ClO_2_	0.81	Not rejected

### Texture

The change in texture of fresh-cut endive leaves after each process variant (Figure 2—right column) was determined directly (day 0), 1 day, and 7 days after treatment for each process extraction point (0–5). For the texture determination, the complete head of lettuce, except the outer leaves, was prepared for analysis and the stem components were not removed. In texture analysis, the required maximum force for the first breakthrough varied between 100 and 150 N, in general. The relatively large deviations at the individual measuring points may be due to the small sample quantity of 10 g or the mixture of hard and soft leaf parts (Stem components were not sorted out). In comparison to unwashed fresh-cut endive (extraction point 0) the washed lettuce became softer, less force was needed. However, compared to the tap water variant (I), no negative (softening) effect of PTW (variant II to V) or ClO_2_ (variant VI) was observed for all process variants at day 0 and 1 (*p* > α). There were moderate variations of texture for the process variant II. A 180 s pre-bathing effected the T [*p* = 0.02 (^*^)] directly after the treatment on day 0. For the process variant III (30 s pre-rinsing, *p* = 0.03) the texture was effected on examination day 1. Undoubtedly, variations of texture due to a sanitizer treatment (see chapter 2.5) have been observed on day 7. The test scenario clearly shows changes in texture, which appear to be highly significant (*p*-value: <0.001 for process variants IV and VI). The results of the statistical evaluation are given in Tables 6–9.

**Table 6:** Statistical evaluation of storage day 0.

**Process variant**	***p*-value**	***H*_**0**_ hypothesis**
Horizontal β evaluation		
I—tap water	0.19	Not rejected
II—PTW	0.02	Rejected
III—PTW	0.74	Not rejected
IV—PTW	0.27	Not rejected
V—PTW	0.98	Not rejected
VI—ClO_2_	0.4	Not rejected
Vertical α evaluation		
Extraction point 5 (EP5)	0.13	Not rejected

**Table 7:** Statistical evaluation of storage day 1.

**Process variant**	***p*-value**	***H*_**0**_ hypothesis**
Horizontal β evaluation		
I—tap water	0.48	Not rejected
II—PTW	0.41	Not rejected
III—PTW	0.03	Rejected
IV—PTW	0.31	Not rejected
V—PTW	0.43	Not rejected
VI—ClO_2_	0.64	Not rejected
Vertical α evaluation		
Extraction point 5 (EP5)	0.39	Not rejected

**Table 8:** Statistical evaluation of storage day 7.

**Process variant**	***p*-value**	***H*_**0**_ hypothesis**
Horizontal β evaluation		
I—tap water	0.001	Rejected
II—PTW	0.99	Not rejected
III—PTW	0.001	Rejected
IV—PTW	<0.001	Rejected
V—PTW	0.06	Not rejected
VI—ClO_2_	<0.001	Rejected
Vertical α evaluation		
Extraction point 5 (EP5)	<0.001	Rejected

**Table 9:** Statistical evaluation over all storage days (day 0, 1, and 7) at EP5.

**Process variant**	***p*-value**	***H*_**0**_ hypothesis**
ANOVA evaluation over day 0, 1, and 7 at EP5		
I—tap water	0.03	Rejected
II—PTW	0.002	Rejected
III—PTW	0.34	Not rejected
IV—PTW	0.53	Not rejected
V—PTW	0.06	Not rejected
VI—ClO_2_	0.25	Not rejected

### Nitrate

Dietary nitrate is mainly derived from the consumption of vegetables, in particular green leafy vegetables (29). Due to their dietary importance, nitrate levels in fresh-cut endive were monitored during storage (Table 10). However, the EU has regulated the maximum levels of nitrate for fresh lettuce with 2,500-4,500 mg NO_3_ kg^−1^ depending on the harvest time and conditions (30). Since PTW, which was used to wash fresh-cut endive, contained both significant concentrations of nitrite and nitrate, it was necessary to determine whether the nitrate content in lettuce increases after washing and exceeds acceptable limits and guidance values. This could be an exclusion criterion for the approval of this innovative washing method. The absorption of nitrate may be increased by the fresh cut edges. In order to determine the nitrate values, IC analysis was used. The IC-samples were prepared immediately after processing (day 0) and the nitrate content in unwashed, tap water, PTW and ClO_2_ washed endive were measured.

**Table 10:** Nitrate concentrations of fresh-cut unwashed and washed endive.

**Sample/process variant**	**Extraction points**						**Nitrate concentration in**
	**0**	**1**	**2**	**3**	**4**	**5**	
Distilled water	0.1 ± 0.09						[mg l^−1^]
Tap water	1.6 ± 0.05						[mg l^−1^]
PTW	2.48 ± 0.56						[mg l^−1^]
ClO_2_	2.0 ± 0.1						[mg l^−1^]
Unwashed	1,793.6 ± 373.7						[mg kg^−1^]
I—tap water		937.0 ± 750.9	758.0 ± 561.6	671.8 ± 478.2	907.2 ± 724.6	860.9 ± 647.4	[mg kg^−1^]
II—PTW		1,013.7 ± 387.3^a^	811.6 ± 181.7	729.1 ± 710.2	635.1 ± 355.7	632.6 ± 350.2	[mg kg^−1^]
III—PTW			709.9 ± 354.2^a^	436.7 ± 319.9	377.9 ± 221.6	403.3 ± 257.6	[mg kg^−1^]
IV—PTW			509.9 ± 185.9	533.9 ± 174.9	1,019.0 ± 489.5^a^	631.2 ± 298.3	[mg kg^−1^]
V—PTW		1,134.2 ± 561.4^a^	891.0 ± 289.4^a^	783.7 ± 556.7	1,206.2 ± 483.8^a^	739.4 ± 291.3	[mg kg^−1^]
VI—ClO_2_			380.6 ± 199.2	539.4 ± 274.3	552.7 ± 189.3^b^	422.6 ± 306.4	[mg kg^−1^]

a *PTW application*.

b *ClO_2_ application*.

*All experiments were repeated three-fold with n = 5 resulting in n = 15*.

The nitrate content of unwashed endive (process variant I) was the highest with 1,793.6 ± 373.7 mg kg^−1^. Within all process variants (I–VI) the nitrate concentration was lower compared to unwashed lettuce. Despite the high water solubility of NO_3_, the detected concentrations of nitrate in the variants I to VI were between 377.9 ± 221.6 mg ${\text{NO}}_{3}^{-}$ kg^−1^ and 1,206.2 ± 483.8 mg ${\text{NO}}_{3}^{-}$ kg^−1^. The concentrations are summarized in detail for each process variant and extraction point in Table 10. For all PTW-application points, an increased ${\text{NO}}_{3}^{-}$ concentration compared to the other extraction points was noticeable. As expected, ClO_2_ did not lead to an increase in ${\text{NO}}_{3}^{-}$. In PTW variants (II–V), the ${\text{NO}}_{3}^{-}$ concentration did not increase compared to unwashed (extraction point 0) and tap water (variant I) washed lettuce at the final extraction point 5. Importantly, all ${\text{NO}}_{3}^{-}$ concentrations were significantly below the maximum permitted value of 2,500 mg ${\text{NO}}_{3}^{-}$ kg^−1^ for iceberg-type lettuce (2,000–2,500 mg ${\text{NO}}_{3}^{-}$ kg^−1^) and for fresh lettuce (except iceberg-type) of 2,500–4,500 mg ${\text{NO}}_{3}^{-}$ kg^−1^ (30).

### SEM

**Figure 3:**
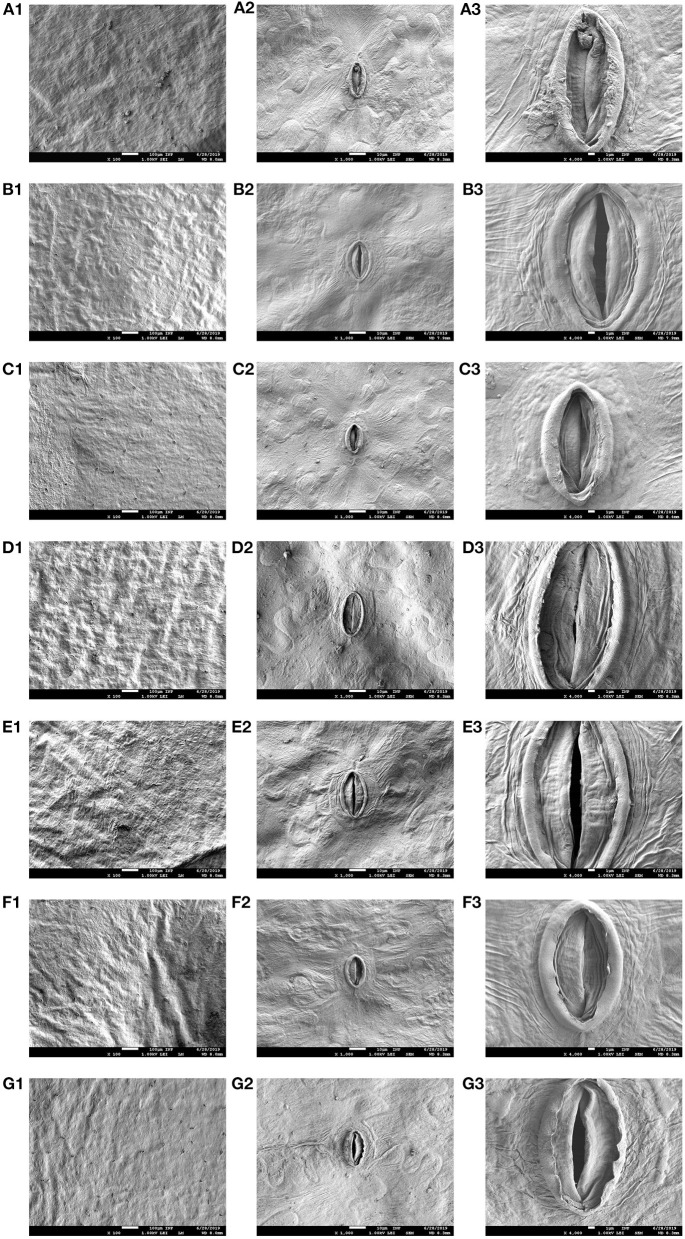
Scanning electron microscopy (SEM) of the fresh-cut lettuce. For each process variant (I–VI), three magnifications are shown: left—×100, middle—×1,000, right—×4,000. The scenarios shown are: **(A)** unwashed (extraction point 0), **(B)** tap water washed (variant I), **(C)** PTW washed—variant II, **(D)** PTW washed—variant III, **(E)** PTW washed—variant IV, **(F)** PTW washed—variant V, and **(G)** ClO_2_ washed (variant VI).

**Figure 4:**
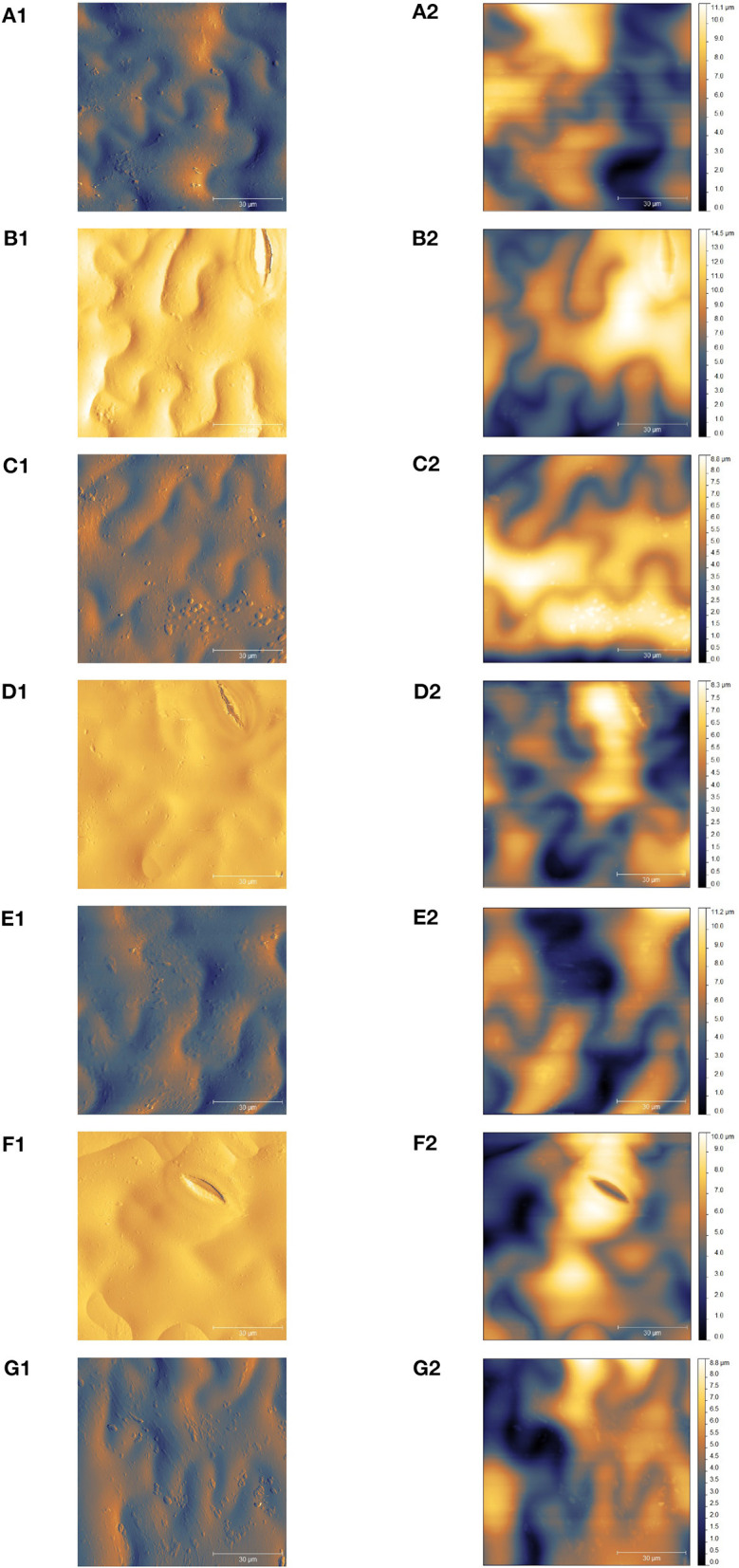
Atomic force microscopy (AFM) of the fresh-cut lettuce. For each process variant (I–VI), two micrographs are shown: left—error signal/deflection micrograph, right—height micrograph. The variants shown are: **(A)** unwashed (extraction point 0), **(B)** tap water washed (variant I), **(C)** PTW washed—variant II, **(D)** PTW washed—variant III, **(E)** PTW washed—variant IV, **(F)** PTW washed—variant V, and **(G)** ClO_2_ washed—variant VI.

Microscopic methods for a deeper characterization of the influence of a PTW on lettuce were chosen. The focus of these investigations was on the food quality of fresh-cut lettuce mirrored by color- and texture analysis. Additionally, SEM and AFM were used to visualize variations on the leaves surface due to a sanitizer treatment. Especially TEM revealed insight into the cell interior and possible changes of cell organelles can be observed. In the texture analysis (see section Texture), only a moderate influence (process variant II, day 0, *p* = 0.02) on the leaf structure directly after washing with PTW was found. This was confirmed by the SEM (Figure 3) and AFM (Figure 4) analysis, were no sever, structural alterations have been observed. Overall, the SEM analysis showed no clearly visible changes between the samples and the unwashed reference. When washing the lettuce with tap water, or PTW (irrespective of the process variant) there was no noticeable influence and intact stomata—both open and closed—were observed in all samples (Figure 3 middle and right column). The temperature of the water in all scenarios was 21.5°C ± 0.1°C. Only after the treatment of lettuce with ClO_2_, does the lettuce surface appear flatter and smoother, i.e., less rough and structured (Figure 3G, magnification × 100). The stomata seemed to be less raised and, more collapsed. In the ×4,000 magnification (Figure 3G), the outer edge of the stomata appeared somewhat attacked.

### AFM

Our investigations showed results of 8–14 μm for the endive leaves in the height micrograph. The wave-like structure, previously observed through SEM analysis, was clearly seen in the error signal micrograph (Figure 4, left column). The slight variations in the height profiles were probably due to the presence of stomata in the scanned section. The slight deviations for ClO_2_ observed in the SEM investigations were not visible in the AFM images. This may be due to the small scanned area in the AFM. On the other hand, the reason could also be that the samples are pre-treated for SEM and not for AFM. The proposed advantage of AFM analysis is in sample preparation, where they were not subject to dehydrating steps and were relatively natively analyzed. AFM was proposed as a good alternative for SEM.

### TEM

**Figure 5:**
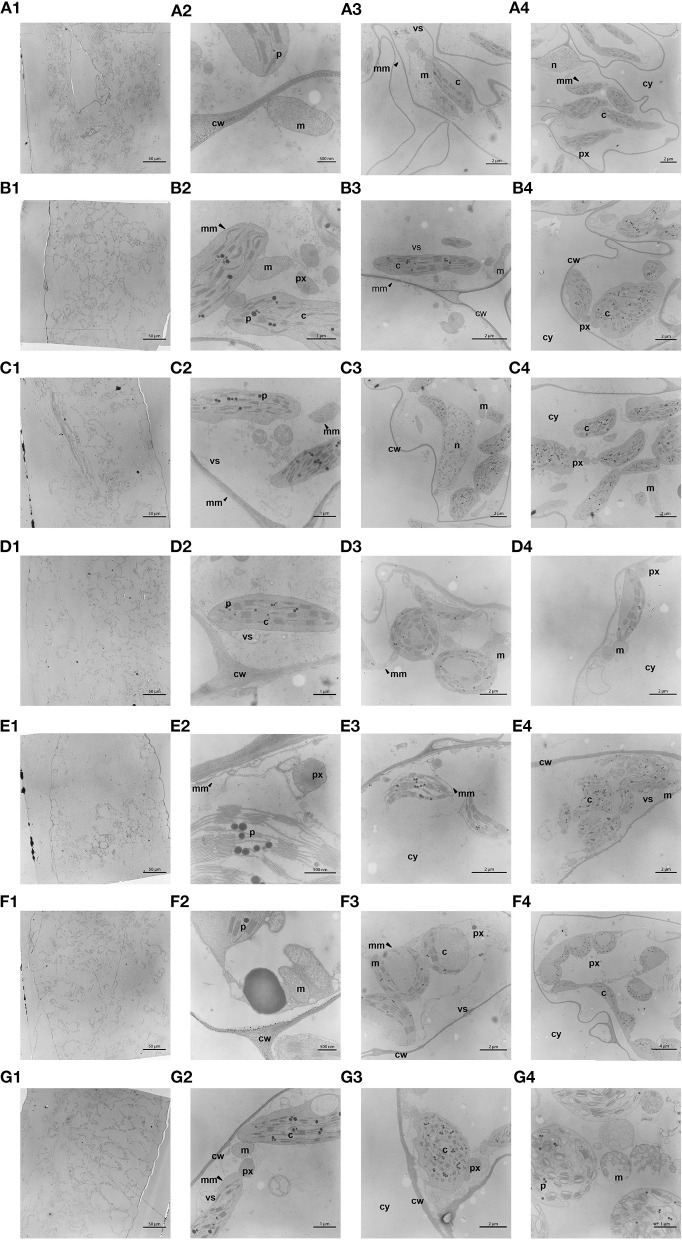
Transmission electron microscopy (TEM) of the ultrastructure of the fresh-cut lettuce leaf tissue. For each process variant, four micrographs are shown: from left to right—overview of the ultrathin section, three times a detailed micrograph of the same section depicting all detected cell organelles. The process variants shown are: **(A)** unwashed (extraction point 0), **(B)** tap water washed (variant I), **(C)** PTW washed—variant II, **(D)** PTW washed—variant III, **(E)** PTW washed—variant IV, **(F)** PTW washed—variant V, **(G)** ClO_2_ washed—variant VI. The organelles are: nucleus (n); cytoplasm (cy); cell wall (cw); chloroplast (c); mitochondrion (m); vesicle (vs); plastogloboli (p); peroxisome (px); membrane (mm; plasmalemma or tonoplast).

The TEM-analysis (Figure 5) of endive leaf tissue from unwashed lettuce showed the typical cell organelles (31–33), with the exception of vacuoles (Figure 5A). The absence of vacuoles was probably an artifact of preparation. Differences in the composition of cell organelles were not found for the applied treatments by comparison with the unwashed reference. However, chloroplasts in leaf tissue showed morphological changes after washing with ClO_2_ (Figure 5G), particularly in the grana the grana (stacks of thylakoid discs). An altered structure of the thylakoid membranes was observed. TEM micrographs of thylakoid membranes clearly revealed the drastic difference in the thylakoid structure in ClO_2_ washed fresh-cut lettuce, where the stacks in grana were attached to one another to form large clearances. Further, the stroma thylakoids were disrupted (Figure 5G). In contrast to the addition of ClO_2_ to the washing water, the use of PTW for lettuce washing had no effect on the grana of the chloroplasts or other organelles, regardless of the application point of PTW (Figures 5C–F).

## Discussion

Food safety should always be considered in combination with retention of food quality characteristics for approval and adoption of emerging technologies, in order to increase acceptance by stakeholders (industry) and consumers and to identify the optimal process variant taking into account individual needs.

Previous publications investigated the antimicrobial effects of plasma processed air (PPA) and this PTW for retention of food safety profiles using different target surfaces and microorganisms (25, 26, 34–38). These studies addressed the decontamination potential of PPA or PTW on microorganisms relevant for safe shelf life extension, on the artificial and natural microbial loads of fresh-cut lettuce, and the scalability of the plasma technology and pilot-scale application. However, the impacts on fresh product quality characteristics represented a research lacuna, which was therefore the focus of the current study.

The investigations on color and texture showed only sparsely negative influences of a PTW or ClO_2_ treatment, which cannot be detected by the used ANOVA. The systematic color screening of treated leaves revealed no statistically significant support of our assumptions (H_1_-hypothesis) and the null-hypotheses cannot be rejected for every single process variant (all *p* > α). On the other hand, statistically meaningful structural changes of product matrix of the fresh-cut endive within the storage trial of 7 days were observed (process variant I, III, IV, and VI; *p*-values < 0.01). The logical construct when the β-null hypothesis (horizontal) and the α-null hypothesis (vertical) must be rejected states there were structural alterations due to a sanitizer treatment, which become obvious on day 7 for process variant I, III, IV, and VI (*p*-value <0.001). Additional, for a storage period of 7 days, structural changes become statistically meaningful for the process variant I (tap water) and the process variant II (*p* < 0.03). However, since the tap water-reference showed a pronounced alteration especially after a storage time of 7 days, changes might attribute to ordinary aging of the biological product matrix. Contrary, ClO_2_ and PTW show statistically meaningful texture alterations after their treatment and a storage of 7 days (*P* > 0.001), but distinct changes from day to day could not be supported by statistics (*p* > α). A picture, which states changes due to a sanitizer treatment, but they do not persist over the whole storage time (*p*-values > α). This results in the question if the sanitizer somehow conserves the leaves after they alter their structural appearance in a certain way. Alternatively, the other way around, do ClO_2_ and PTW trigger aging processes in the leaves, which lead to massive structural changes after a 7-day storage period? Nevertheless, future investigations with separated lettuce leaf components might provide insight in such processes and support the testing of distinct hypotheses. It is important to have statistically meaningful basic values (e.g., alterations in color and texture) as a basis for logical links answering more holistic questions. Consequently, these quality characteristics are retained using this emerging technology.

Lettuce accumulates nitrate in its leaves during its growth (39, 40). Among the foods consumed by humans, plants represent between 72 and 94% of daily intake of nitrate (41). Dietary nitrate is mainly derived from the consumption of vegetables, in particular green leafy vegetables such as rocket (4,800 mg ${\text{NO}}_{3}^{-}$ kg^−1^) and lamb's lettuce (2,130 mg ${\text{NO}}_{3}^{-}$ kg^−1^) (29). However, nitrate contents in fresh-cut endive can decrease during storage (17), due to a concomitant growth of nitrate-metabolizing microorganisms (29, 42). If nitrate is converted to nitrite, this can have negative health effects, as nitrite can be a source of carcinogenic nitrosamines (43–45). Therefore, the European Union established the maximum permissible levels from 4,000 to 5,000 mg ${\text{NO}}_{3}^{-}$ kg^−1^ for the winter season and 3,000 to 4,000 mg ${\text{NO}}_{3}^{-1}$ kg^−1^ for the summer season (30). The endive samples examined in all our process variants remained within the legally prescribed nitrate values. Therefore, the use of PTW as a washing additive maintained endive food quality concerning the nitrate content. Further investigations about other valuable content compounds such as vitamin C may be also useful.

The promising results for food quality retention of color, texture, and nitrate obtained using PTW as process water additive were supported by the microscopic measurements based on SEM, AFM, and TEM. For trials using tap water or PTW, there was no significant difference noted in the influence compared to each other and to unwashed endive samples. However, for the application of ClO_2_ the thylakoid stacks seem to be affected in TEM images, therefore the light-dependent reactions of photosynthesis may be negatively influenced as the interval between photosystem II complexes was expected to be larger. Although harvested and processed lettuce no longer has to undergo photosynthesis, it is still a living tissue, which may be subject to an accelerated aging process due to the change. This may not only affect the shelf life but also the quality of the tissue. Fujii et al. (46) mentioned in their work on photo inhibition of chloroplast genome-modified and common lettuce, that not only were the grana altered, but also that the plastoglobuli, which contain accumulated lipids and lipoproteins, were enlarged (46).

The use of PTW as a new washing and sanitization treatment provides a means to extend safe shelf life while maintaining produce food quality characteristics within regulatory guidelines. To fulfill all requirements of a regulatory environment, further investigations on sensory properties as well as toxicology are necessary, notwithstanding the need to examine marketability and cost benefit with the a product life cycle analysis.

In conclusion, the present study demonstrated that PTW could be used as a process water agent to improve conventional washing methods in fresh-cut processing at diverse stages of a process line, without any impairment of the quality of fresh-cut endive directly after treatment and during subsequent storage for 7 days. The promising results and the advantages of PTW including low-temperature, simple and cheap generation and demonstrated comparability to current procedures such as tap water rinsing and chlorinated water offer a wide range of innovative applications.

## Data Availability Statement

The raw data supporting the conclusions of this article will be made available by the authors, without undue reservation.

## Author Contributions

US and PB: conceptualization. US, TW, CW, JSc, and HB: methodology. US, OH, HW, TW, CW, JSc, and HB: investigation. US: writing–review and editing. OH, DB, PB, and JE: writing–review and editing. JSt: visualization. DB, PB, and JE: supervision. JE: project administration and funding. All authors have read and agreed to the published version of the manuscript.

## Conflict of Interest

The authors declare that the research was conducted in the absence of any commercial or financial relationships that could be construed as a potential conflict of interest.
